# Artificial intelligence in the field of pharmacy practice: A literature review

**DOI:** 10.1016/j.rcsop.2023.100346

**Published:** 2023-10-21

**Authors:** Sri Harsha Chalasani, Jehath Syed, Madhan Ramesh, Vikram Patil, T.M. Pramod Kumar

**Affiliations:** a Dept. of Pharmacy Practice, JSS College of Pharmacy, JSS Academy of Higher Education & Research, Mysuru 15, Karnataka, India; b Dept. of Radiology, JSS Medical College & Hospital, JSS Academy of Higher Education & Research, Mysuru 15, Karnataka, India; c JSS College of Pharmacy, Mysuru, Karnataka, India

**Keywords:** Pharmacy practice, Clinical pharmacist, Artificial intelligence, Pharmacointelligence

## Abstract

Artificial intelligence (AI) is a transformative technology used in various industrial sectors including healthcare. In pharmacy practice, AI has the potential to significantly improve medication management and patient care. This review explores various AI applications in the field of pharmacy practice.

The incorporation of AI technologies provides pharmacists with tools and systems that help them make accurate and evidence-based clinical decisions. By using AI algorithms and Machine Learning, pharmacists can analyze a large volume of patient data, including medical records, laboratory results, and medication profiles, aiding them in identifying potential drug-drug interactions, assessing the safety and efficacy of medicines, and making informed recommendations tailored to individual patient requirements. Various AI models have been developed to predict and detect adverse drug events, assist clinical decision support systems with medication-related decisions, automate dispensing processes in community pharmacies, optimize medication dosages, detect drug-drug interactions, improve adherence through smart technologies, detect and prevent medication errors, provide medication therapy management services, and support telemedicine initiatives.

By incorporating AI into clinical practice, health care professionals can augment their decision-making processes and provide patients with personalized care. AI allows for greater collaboration between different healthcare services provided to a single patient. For patients, AI may be a useful tool for providing guidance on how and when to take a medication, aiding in patient education, and promoting medication adherence and AI may be used to know how and where to obtain the most cost-effective healthcare and how best to communicate with healthcare professionals, optimize the health monitoring using wearables devices, provide everyday lifestyle and health guidance, and integrate diet and exercise.

## 1 Introduction

Alan Turing's seminal work, “Computing Machinery and Intelligence,” published in 1950, marked the beginning of the artificial intelligence (AI) debate.^1^ In 2004, John McCarthy defined AI as “the science and engineering of making intelligent machines, especially intelligent computer programs.”.^2^

AI has emerged as a transformative technology that has revolutionized a wide range of industries worldwide. From finance to healthcare, manufacturing, and transportation, AI has been at the forefront of innovation, enabling previously inconceivable advances. AI has paved the way for unprecedented automation, efficiency, and decision-making capabilities by leveraging intelligent algorithms, machine learning (ML), and data analytics.

### 1.1 Artificial intelligence in healthcare

AI in healthcare has evolved dramatically over the last five decades, leading to significant advancements in a variety of medical fields.^3^ The introduction of ML and deep learning (DL) has expanded AI applications, enabling personalized medicine rather than relying solely on algorithms. AI has significantly impacted clinical decision-making, disease diagnosis, as well as clinical, diagnostic, rehabilitative, surgical, and predictive practices.^4^

This advancement in AI technology has paved the way for improved diagnostic accuracy, streamlined provider workflow, improved clinical operation efficiency, disease, and therapeutic monitoring, precise procedures, and, ultimately, better patient outcomes.^5^^,^^6^

### 1.2 Pharmacy practice

Pharmacy practice is an integral part of the healthcare system, which ensures safe and effective medication management and optimized patient care, through various activities such as medication reconciliation, medication review, medication therapy management (MTM), providing drug information, patient education, adverse drug reaction (ADR) monitoring and interprofessional collaborations.^7^

With rapid advancements in the healthcare sector, the number of prescriptions, complex drug regimens, and administrative tasks has increased noticeably. As a result, there is an increasing demand for advanced technological solutions that can assist healthcare professionals in their daily responsibilities and optimize healthcare service delivery.^8^

The incorporation of AI technologies provides pharmacists with tools and systems that help them make accurate and evidence-based clinical decisions. By using AI algorithms and ML, pharmacists can quickly analyze large amounts of patient data, including medical records, lab results and medication profiles. This allows them to identify potential drug-drug interactions, assess the safety and efficacy of medicines, and make informed recommendations tailored to individual patients.^3^^,^^8^^,^^9^

The application of AI in various areas within the field of pharmacy practice has shown promising prospects. However, existing research gaps need to be addressed to harness the complete potential of AI technologies. The most important aspect is the comprehensive implementation of AI services within existing pharmacy systems and understanding its impact on health and economic outcomes. In this review, we will be exploring the various AI applications in the field of pharmacy practice; the research gaps and challenges; and highlighting the future directions for research within the field.

## 2 Methods

To identify topics of interest for this narrative review, the various databases (PubMed, Google Scholar, and Scopus) were searched for relevant articles. Various search terms were used to identify the relevant literature, which included “Artificial intelligence,” Adverse drug reaction,” “ADR,” “Machine learning,” “Deep learning,” “Neural networks,” “Clinical decision support systems,” “Medical Order Entry Systems,” “Computerized Provider Order Entry,” “Pharmacy practice,” “Clinical pharmacy,” “Community pharmacy,” “Hospital pharmacy,” “Pharmacist,” “Medication therapy management,” “Drug dispensing,” “Medication reconciliation,” “Medication adherence,” “Medication optimization,” “Pharmaceutical care,” “Precision medicine.” The reference list of the relevant articles was also reviewed to identify potentially important papers pertaining to the topic. Two authors independently conducted the search and the most appropriate ones were included into the review.

## 3 Results

### 3.1 AI in pharmacy practice

#### 3.1.1 Adverse drug reaction (ADR) detection

AI has been utilized in several studies for ADR prediction and detection. One such study conducted by Mohsen and colleagues, which combined two distinct datasets: drug-induced gene expression profiles from the Open Toxicogenomics Project-Genomics Assisted Toxicity Evaluation Systems (TG-GATEs) database and ADR occurrence data from the Food and Drug Administration (FDA) Adverse Events Reporting System (FAERS) database in conjunction with Deep Neural Networks (DNN) for ADR prediction. It includes data filtering and cleaning, feature selection, and hyperparameter tuning.^10^

Yalçn et al. developed a ML-based clinical decision support tool (risk score) that predicts whether the identified ADRs would occur by integrating the severity with neonatal adverse event severity scale (NAESS) and probability with the ‘Du'ADRs algorithm into the risk matrix analysis performed by a multidisciplinary team that included a clinical pharmacist. Decision tree induction, a ML method, was used by Hammann et al. to determine the chemical, physical, and structural properties of compounds that predispose them to cause ADRs. For allergic, renal, CNS, and hepatic ADRs, the models had high predictive accuracies (78.9–90.2%).^11^^,^^12^

In a study by Cami et al., a logistic regression classifier to predict unknown ADRs for marketed drugs using structural properties of the drug-ADR network as well as chemical and taxonomic properties of drugs as features was developed.^13^ Rahmani et al. used a random walk algorithm to predict unknown ADRs in a network with drug and ADR nodes, where drug-ADR edges represent known ADRs and drug-drug edges indicate drug target similarity, but they did not validate new ADRs in any real-world clinical data.^14^ Bresso et al. also created a database of the drug, ADR, and target knowledge and used decision trees and inductive logic programming to predict ADR profiles (rather than individual ADRs), which they validated using FAERS.^15^

Bean et al. created a knowledge graph with four different types of nodes: drugs, protein targets, indications, and adverse reactions. Using this graph, they created a ML algorithm based on a simple enrichment test and demonstrated how well this method performs at classifying known causes of adverse reactions.^16^

Furthermore, other studies involved classifying approved drugs from withdrawn drugs to reduce adverse drug effects, extracting adverse drug events (ADEs) from clinical narratives and automating pharmacovigilance, predicting and preventing adverse drug reactions at an early stage to improve drug safety, identifying medications, adverse drug effects, and their relationships with clinical notes, identifying adverse drug effect symptoms and drugs in clinical notes, detecting adverse drug reactions, and detecting ADEs.^17^, ^18^, ^19^, ^20^, ^21^, ^22^, ^23^

Overall, these studies highlight the broad range of AI applications in ADR detection, involving prediction models to clinical decision support tools and knowledge graph-based algorithms.

#### 3.1.2 Clinical decision support system (CDSS)

A clinical decision support system (CDSS) is designed to improve healthcare delivery by supplementing medical decisions with targeted clinical knowledge, patient information, and other health data. Individual patient characteristics are matched to a computerized clinical knowledge base in a CDSS, and patient-specific assessments or recommendations are then presented to the clinician for a decision. This technology enables pharmacists to sift through data and intervene to prevent medication errors, reduce patient complications, and save money.^24^^,^^25^

#### 3.1.3 Community Pharmacy

Healthcare systems are rapidly transitioning from a single hospital-based care module to a collaborative care system based in the community. Pharmacists can help to improve patient safety and efficacy of pharmacotherapy from the hospital to the community. The “robotic dispensing system” in the community pharmacies prepares prescribed medicines. It consists of three parts^26^:.

(1) An automated dispensing robot operated by pharmacy support staff,
(2) An automated dispensing robot for powdered medication, and
(3) A bar-coded medication dispensing support system with personal digital assistance.

ML models also allow e-mails to be personalized faster and more accurately than any human. Chatbots can be used to improve service delivery efficiency.^8^ Chatbots can simulate interactions between customers and customer service representatives. Chatbots can automatically resolve customer complaints and queries, and difficult questions are routed to human staff. Chatbots in community pharmacies can be programmed to simulate interactions between pharmacists and patients.^27^

Walgreen collaborated with a telehealth company, to develop a video chat platform for patients to interact with healthcare professionals.^28^ AI can also help with inventory management, where community pharmacists can predict what their patients will require in the future, stock them, and use personalized software to send e-mails to remind patients of drug requirements. A patient's future drug purchase can be predicted using AI-powered data analytics. The pharmacist will be able to make better stock procurement decisions if AI can predict the patient's drug purchase.^8^^,^^29^

An AI company, created a software for a German online and catalog retailer, which can predict what the retailer will sell in 30 days with 95% accuracy. This resulted in reduction in delivery schedule for purchased products from one to two days by allowing direct delivery from the supplier to the consumer without passing through the warehouse.^30^

The University of California San Francisco (UCSF) Medical Center also prepares and tracks medications using robotic technology. They claim that the technology has prepared 3,500,000 medication doses without error. The robot has proven to be far superior to humans in terms of both size and ability to deliver accurate medications. The robotic technology's capabilities include the preparation of oral and injectable medicines, including toxic chemotherapy drugs. The robotics package, and dispense individual doses of pills. The machines also assemble the doses onto a bar-coded plastic ring, which contains all medications that a patient must take within 12 h. The automated system's capabilities include the ability to prepare sterile preparations for chemotherapy as well as fill intravascular syringes with the appropriate medications.^31^

#### 3.1.4 Computerized prescriber order entry (CPOE)

Medication errors, according to the Institute of Medicine, are the most common type of error in healthcare, accounting for approximately 7000 deaths each year.^32^ Although there are numerous causes of medication errors, published research estimates that 11.4% of these errors are directly related to drug name mismatches, such as illegible prescriptions, confusing dosage forms, and misunderstood abbreviations.^33^

Computerized Physician Order Entry (CPOE), also known as Computerized Provider Order Entry or Computerized Practitioner Order Entry, is a process by which a physician enters and sends medication orders and treatment orders, as well as laboratory, admission, radiology, referral, and procedure orders electronically through a computer application, rather than using traditional methods such as paper charts, verbal orders, telephone, and fax. This method reduces errors caused by illegible handwriting or transcription errors in medication instructions.^34^

These CPOE systems control the selection, display, and storage of medication histories and the electronic transmission of medication orders to dispensing pharmacists and pharmacies. This new paradigm offers numerous opportunities to protect patient safety (e.g., allergy or renal dosing alerts), but also raises the possibility of many new types of predictable and unpredictable prescribing and dispensing errors.^35^

#### 3.1.5 Dose recommendations

Patients can benefit from a personalized AI/ ML-based dosage recommendation system that incorporates data from multiple sources, such as safety and effectiveness metrics, electronic health records, disease details, treatment history, and patient feedback. These systems aim to improve treatment efficacy while minimizing side effects. Reinforcement learning algorithms have shown promise in predicting and adjusting dosages for precision-based cancer treatment.^36^

The most recent innovation with the potential to improve chronic disease care is a novel dosing optimization system, which is a platform for actionable dosing optimization that was created to improve chemotherapy dosing precision. The algorithm considers treatment response over time, predicting dosing requirements dynamically to maintain required efficacy and safety levels.^37^

##### 3.1.5.1 AI in high-risk drug dosing

Because of the dynamic profile of patients receiving the drug, optimizing vancomycin therapy remains a challenge in current clinical practice. Many factors, including renal function, concomitant drugs, and weight, are known to influence vancomycin dose-concentration response. Various approaches, such as dosing nomograms and Bayesian estimation methods, have been used in clinical practice to guide clinicians in vancomycin dosing.^38^^,^^39^ Wang Z, Ong CL, and Fu Z created a new AI-assisted dosage titration approach that has the potential to improve on traditional approaches. This approach is especially useful for guiding decision-making for inexperienced doctors in making consistent and safe dosing recommendations for high-risk medications like vancomycin.^39^

Researchers have also developed prediction models for the dosage of drugs like digoxin^40^ and warfarin,^41^ aiding in avoiding ADEs from dosage errors.

#### 3.1.6 Drug-drug interactions

Drug-drug interactions (DDIs) have been identified as a significant cause of ADRs, which contribute to rising healthcare costs.^42^^,^^43^ Predicting DDI necessitates the use of multiple drug characteristics and known DDI. The most used databases are DrugBank,^44^ SIDER,^45^ TWOSIDES,^46^ Kyoto Encyclopedia of Genes and Genomes (KEGG),^47^ Lexicomp,^48^ and Micromedex.^49^

Existing DDI computational methods are classified into three types: similarity-based methods, networks-based methods, and ML methods. Van Laere et al. developed an algorithm that predicts QTc prolongation and issues alerts when DDIs increase the risk of QTc prolongation.^50^ Suyu Mei and Kun Zhang proposed a simple f-drug target profile representation to depict drugs and drug pairs, which was used to build an l2-regularized logistic regression model to predict DDIs.^43^

Song et al. created a largescale DDI predictor by combining five types of drug similarities: 2D molecular structure similarity, 3D pharmacophoric similarity, drug interaction profile similarity, target similarity, and adverse effect similarity, and provided a Polynomial Kernel Support Vector Machines (PK-SVM) classifier to carry out the predictive work.^51^

#### 3.1.7 Electronic health record (EHR)

The implementation of a new predictive EHR algorithm can lead to improved clinical decisions through software can detect and alert, when a prescribed drug appears to deviate from its pattern of appropriate use by using large amounts of EHR data and AI to learn patterns concerning appropriate medication use. Furthermore, AI could aid in drug selection decisions by indicating which patients are unlikely to experience adverse effects from a specific drug via automated classification.^52^^,^^53^ Patient Safety Learning Laboratory (PSLL) embedded AI into the EHR systems can identify, assess, and mitigate threats to patient safety.^54^

The use of natural language processing (NLP) and ML in hospital and health system pharmacies to access and analyze unstructured, free-text information captured in millions of EHRs (e.g., medication safety, patients' medication history, adverse drug reactions, interactions, medication errors, therapeutic outcomes, and pharmacokinetic consultations) may become an essential tool to improve patient care and perform real-time evaluations of the efficacy of medications. This strategy has enormous potential to support risk-sharing agreements and guide decision-making in pharmacy and therapeutics (P&T) Committees.^55^

Similar model was developed by Balestra M et al., a predictive model for flagging orders requiring intervention using only information about the ordering provider's interaction with the EHR.^56^

#### 3.1.8 Identification of potentially inappropriate drug

Potentially inappropriate medications (PIMs) are medications whose risks outweigh the benefits when administered to patients.^57^ The prevalence of comorbid conditions and polypharmacy among elderly patients puts them at risk of potentially inappropriate prescribing (PIP). There are currently several criteria for assessing PIP, including the Beers criteria^58^ and the STOPP/START criteria.^59^ Despite the fact that these criteria are widely used for post-event evaluation. However, by detecting PIP early, physicians and pharmacists will be able to identify patients at risk of PIP and implement individualised interventions to reduce the risk of ADR. Several AL/ML algorithms are increasingly being used to develop predictive models for PIMs prescription.^60^

Chun-Tien Tai and colleagues predicted the risk of digoxin treatment using ML. The results demonstrated that the best model performance successfully identified the risk. This study found that ML techniques can improve prediction accuracy for high alert drug (HAD) medication treatment, lowering the risk of ADEs, and improving medication safety.^61^ Wongyikul et al. created a HAD screening protocol with a ML model that used Gradient Boosting Classifier and screening parameters to identify HAD prescription errors from outpatient and inpatient drug prescriptions. The ML algorithm identified over 98% of actual HAD mismatches in the test set and 99% in the evaluation set when screening drug prescription events with a risk of HAD inappropriate use. This study demonstrated that ML played an important role in screening and reducing errors in HAD prescriptions.^62^

Patel et al. developed predictive models using ML algorithms to identify predictors of inappropriate use of nonsteroidal anti-inflammatory drugs (NSAIDs) of PIP in elderly patients with osteoarthritis.^63^

Xingwei et al. used five sampling methods, three feature screening methods, and eighteen ML algorithms to handle process data and establish risk warning models for potentially inappropriate prescriptions for elderly patients with cardiovascular disease. The study enrolled 404 patients, 318 (78.7%) with PIP, 112 (27.7%) with PIMs rate, and 273 (67.6%) with potential prescribing omissions errors (PPO). Following data sampling and feature selection selecting characteristics, 15 datasets were obtained, based on which 270 risk warning models were built to predict PIP, PPO, and PIM, respectively. The study results found the important factors in the PIP risk warning model to be angina, the number of drugs, the number of diseases, and age. The risk warning platform built was able to predict PIP, PIM, and PPO with acceptable accuracy, predictive performance, and clinical application potential.^60^

#### 3.1.9 Medication adherence

Approximately half of patients with chronic diseases do not take their medications as prescribed, resulting in increased morbidity and mortality; and costing an estimated 100 billion USD per year.^64^

Although pharmacist-led interventions appear to be the most effective in promoting medication adherence, they are frequently complex, involving multiple healthcare providers and multiple components. Since medication adherence barriers are complex and varied, solutions to improve adherence must be multifactorial, and AI technology may be viewed as a promising aspect of such interventions.^65^

There are various AI technologies used for promoting and monitoring medication adherence. Based on their technical designs and adherence monitoring functions, the identified technology types were divided into eight major groups: electronic pillboxes or bags, electronic pill bottles, ingestible sensors, blister pack technology, electronic medication management systems, patient self-report-based technology, video-based technology, and motion sensor technology.

##### 3.1.9.1 The medication event monitoring system (MEMS)

A sensor embedded in the pill cap allows the MEMS to record every time the patient opens the pill bottle. Some newer electronic pill bottle technologies can wirelessly transmit patient medication adherence data, allowing for real-time assessment and monitoring of patient medication adherence.^66^

*Nearfield communication (NFC)* capabilities are frequently built into newer smartphones and medical devices, which can simplify the workflow of patient self-monitoring. NFC is a short-range communication standard that allows data transmission between two NFC devices within a few centimeters (touching). NFC tags can be used to track medication adherence. Patients can track their medication intake by bringing such NFC tags into contact with a smartphone.^67^

Special blisters can be used to track medication intake via NFC. These smart blisters are protected by a foil that contains an electronic circuit. When the tablets or capsules are removed from the blister, a microcontroller detects the interruptions in the conductive paths and records the time and date.^67^^,^^68^ eDispensers, which can both remind patients to take their medications and directly provide them with them.^69^

##### 3.1.9.2 Motion sensor technology

Other methods are using triaxial accelerometers in wireless wearable devices to record and analyze the patient's hand movements. The addition of a fluorophore to the medication, which can be detected in the bloodstream with a monitoring device on the patient's wrist.^70^^,^^71^

*Ingestible sensors*, also referred to as digital pills or digital ingestion monitoring, are a technological system that consists of microsensors, an adhesive external monitor worn on the abdomen, and a mobile app. The medication and micro-ingestible sensors are co-encapsulated and ingested into the body, where stomach gastric fluids dissolve the capsule containing the medication and sensor. When the sensor detects gastric fluid, it sends a unique signal to the external monitor. The detected ingestion event is sent to a mobile app, which uploads the event's date and time stamp, as well as other recorded physiological measures (for example, heartbeat), to a central server.^66^

##### 3.1.9.3 Electronic medication management systems (EMMS)

The radio frequency identification (RFID)-based medication adherence intelligence system is also available for monitoring medication adherence.^72^, ^73^, ^74^

##### 3.1.9.4 Video-based monitoring technology

Most video-based adherence monitoring technologies use video cameras to allow patients to self-record medication ingestion event videos, which are retrospectively analyzed by HCPs or, AI. *Patient Self-reporting Technology*, like EMMS, differ in their specific functionalities, but they all collect subjective medication adherence data by interacting with the patient via phone calls, smart buttons, eDiaries, web-based platforms, and mobile apps. For most self-reported devices, patient adherence is available in real-time.^66^

#### 3.1.10 Medication errors identification

The Food and Drug Administration (FDA) receives over 100,000 reports from the United States each year regarding suspected medication errors (MEs).^75^ Prescription errors occur at rates ranging from 0.3 to 9.1% in European hospitals, while dispensing errors occur at rates ranging from 1.6 to 2.1%.^76^ According to reports, a comprehensive and systematic approaches to patient safety can prevent up to 70.2% of ME-related harm. Implementation of electronic prescription systems, robust medication error surveillance, and the use of barcode medication administration systems are promising strategies for reducing MEs occurrence.^77^

An Israel based company was first to launch a commercial system that uses ML techniques to prevent prescription errors. This system detects overdose and underdose prescriptions with low false-positive rates by analyzing EHRs and generating automatic alerts.^78^ Segal et al. evaluated the utility of a ML-based CDSS in clinical practice. The system examined 78,017 prescriptions, generated 282 alerts (0.4%), and resulted in the discontinuation or modification of 135 prescriptions.^79^

Santos H. et al. proposed an unsupervised method for detecting potential outlier prescriptions called density-distance-centrality (DDC). A dataset of 563 thousand prescribed medications was used to compare the proposed approach to various state-of-the-art outlier detection techniques. In comparison to other methods used to solve this problem, the approach achieves better results in the task of detecting overdose and underdose in medical prescriptions in the experiments. Furthermore, most of the false positives detected by the algorithm were potential prescription errors.^80^ A software as a service (SaaS) system that uses AI to assist clinical pharmacists in decision-making, was developed to improve patient outcomes.^81^ Nagata et al. used ML to create an algorithm for detecting prescription errors in overdoses and underdoses.^82^

Similarly, Yalçin N. et al. developed a model that predicts MEs detected by the clinical pharmacist during the pharmacotherapy process (prescription, preparation, administration, and monitoring) of patients admitted to the NICU using a newborn-centered approach (ML algorithms). The goal was to reduce physician and nurse workload while preventing MEs as part of pharmacotherapy optimization.^83^

A French company launched a hybrid AI decision support system in a typical hospital setting, which combined ML and a rule-based expert system to predict medication errors at the patient level rather than at the level of individual prescription orders.^84^

#### 3.1.11 Medication therapy management (MTM)

The comprehensive medication management (CMM)-Wrap program used a novel AI platform that combines population health and telemedicine to identify and prioritize at-risk members and provide AI decision support for interventions using robust data collection and reporting as well as proprietary MedRiskScores (risk scores). This CMM-Warp involved a disease therapy management provider, combining population health and telemedicine to identify and prioritize the patients with increased risk. They provided remote telephonic services by teams of disease management-trained medical assistants and clinical pharmacists. The research results shown that when pharmacists and medical assistants who have received appropriate training work together with advanced AI systems to deliver CMM services over the phone, led to a decrease in healthcare expenses and a reduction in emergency department visits and hospital admissions. These positive outcomes can be considered potential signs of enhanced well-being.^85^

During the COVID-19 pandemic, a grade 3 A specialized hospital in Shanghai, launched an AI-based internet hospital pharmacy service.^86^ The prescription rules were developed and embedded into the internet hospital system to review the prescriptions using AI, after which the pharmacists would review and the medications would be dispensed after a double check. Then, a “medicine pick-up code” is generated, which is a Quick Response (QR) code that represents a specific offline self-pick-up order (fragile drugs, high-risk drugs, and drugs requiring special management and storage at 2–8 °C). Other drugs that could be delivered were entrusted to a third-party pharmaceutical company. Patients or volunteers could retrieve medications from an offline hospital or drugstore by scanning the QR code through the window and waiting for the dispensing machine or pharmacist to dispense the drugs. They also provided medication consultation services, where a volunteer team of licensed pharmacists with extensive clinical experience provided free medication consultation services online.^86^

#### 3.1.12 Telehealth

Telehealth, also known as telemedicine, is the use of medical information exchanged between sites via electronic communication to improve health outcomes.^87^

Chatbots can speed up and simplify history taking by using NLP to provide prompts and questions based on patient responses, such as self-reporting symptoms, and can provide possible diagnoses, including ADE detection, that can be coded and applied to future patient visits.^88^

A conversational AI platform that complies with the Health Insurance Portability and Accountability Act, developed an adverse event (AE) detection module that uses deep learning and NLP via a virtual assistant to recognize and differentiate between different AEs based on the questions and phrases presented. Once the AE has been identified, the module will automatically transcribe and export the information to the pharmaceutical company, as well as assist with FDA reporting.^89^

In telehealth settings, AI has the potential to improve pharmacovigilance. One study found that using automated phone calls to contact patients starting new medications helped to identify ADEs. Patients whose responses indicated the possibility of ADEs were referred to a pharmacist for further assessment. AI could be used to predict which patients should be screened and when they should be contacted. This, in conjunction with other technologies such as patient portals and texting, has the potential to improve the efficiency and effectiveness of pharmacovigilance efforts.^90^

Patients benefit from health information technologies such as telemonitoring, mobile health applications, and wireless monitoring devices. Monitoring data, disease information, symptom diaries, medication logs, reminders, nutrition diaries, and communication tools are examples of these. Wearable devices and mobile health apps can monitor personal analytics, physical status, and physiological parameters, which can help with medication schedules. Patients use networked medical devices ranging from consumer products such as Fitbit and Apple Watch to wearable external devices such as portable insulin pumps and internally embedded devices such as pacemakers. Providers can assess real-time dynamic data generated by wearable devices using software applications on various devices.^88^^,^^91^

The summary of findings is shown in Table 1.

**Table 1:** Summary of findings.

Adverse drug reaction (ADR) detection					
Author, Year	Country	Objective of the Study	Study design	Participants	Main findings
Mohsen A, 2021^10^	Japan	Using various machine learning methods, estimating the likelihood of adverse drug reactions or events (ADRs) during drug discovery.	Database study	Open TG-GATEs (Toxicogenomics Project-Genomics Assisted Toxicity Evaluation Systems) for drug-induced gene expression profiles and FAERS (FDA [Food and Drug Administration] Adverse Events Reporting System) database for ADR occurrence information	A total of 14 predictive models were built using this framework and Deep Neural Networks (DNN), with a mean validation accuracy of 89.4%, indicating that the approach successfully and consistently predicted ADRs for a wide range of drugs. As case studies, researchers looked at how prediction models performed in the context of Duodenal ulcer and fulminant Hepatitis, highlighting mechanistic insights into those ADRs. The developed predictive models will aid in assessing the likelihood of ADRs when testing new pharmaceutical compounds.
Yalçın N, 2022^11^	Turkey	The primary goal of this study was to generate objective risk categories by incorporating severity with NAESS and probability with the ‘Du'ADRs algorithm into the risk matrix analysis performed by a multidisciplinary team that included a clinical pharmacist. The next goal was to create a machine learning-based clinical decision support tool (risk score) that predicts whether these identified ADRs will occur.	Prospective cohort study	The study included all admitted neonates, but those with preexisting hepatic or renal impairment were excluded.	Enoxaparin, dexmedetomidine, vinblastine, dornase alfa, etoposide/carboplatin, and prednisolone were identified as high-risk drugs. According to the random forest importance criterion, the independent variables included in the risk score to predict ADR presence were: systemic hormones (2 points), cardiovascular drugs (3 points), circulatory system diseases (1 point), nervous system drugs (1 point), and parenteral nutrition treatment (1 point) (cut-off value: 3 points). This risk score correctly classified 91.1% of the test set observations (c-index: 0.914).
Hammann, F, 2010^12^	Switzerland	To conduct a comprehensive survey of ADR reports for a wide range of clinically used drugs and to develop computational models for understanding and predicting such reactions	Database study	Structure-activity relationship analysis of adverse drug reactions (ADRs) in the CNS, liver, and kidney, as well as allergic reactions, for a wide range of drugs (*n* = 507) from the Swiss drug registry	For allergic, renal, CNS, and hepatic ADRs, the models had high predictive accuracies (78.9–90.2%). The feasibility of predicting complex end-organ effects using simple models that do not require expensive computations and can be used for (i) compound selection during the drug discovery stage, (ii) understanding how drugs interact with target organ systems, and (iii) generating alerts in post marketing drug surveillance and pharmacovigilance.
Cami A, 2011^13^	USA	(i) To create a predictive approach that integrates various data types, such as structural network properties, drug intrinsic properties, and drug and adverse drug event (ADE) taxonomies, and introduces several previously unexplored covariates. (ii) Using a simulated prospective approach, evaluate network-based predictive models.	Prospective evaluation	Based on a snapshot of a widely used drug safety database from 2005, drug-ADE associations were created for 809 drugs and 852 ADEs and supplemented these data with additional pharmacological information.	The proposed model had an AUROC (area under the receiver operating characteristic curve) statistic of 0.87, with a sensitivity of 0.42 and a specificity of 0.95. These findings imply that predictive network methods can be used to forecast unknown ADEs.
Rahmani H, 2016^14^	Not mentioned	To develop a novel network approach for ADR prediction, called Augmented Random-WAlk with Restarts (ARWAR).	Database study	The side-effects of each drug in the DrugBank database were extracted from the SIDER (Side Effect Resource) database, and 146 drugs with at least 5 target proteins and 5 side-effects were chosen.	According to the empirical results, the ARWAR method outperformed the existing network approach by 20% in terms of average Fmeasure. ARWAR was also capable of generating novel hypotheses about drugs in terms of novel and biologically meaningful ADR.
Bresso E, 2013^15^	France	To develop a method for identifying and characterizing side-effect profiles (SEPs) shared by several drugs.	Database study	Drug annotations from SIDER and DrugBank databases	Cross-validation and direct testing with new molecules were used to assess learning efficiency. A comparison of two machine-learning techniques: decision trees and inductive logic programming. Demonstrated that the inductive-logic-programming method was more sensitive than decision trees and could successfully exploit background knowledge such as functional annotations and drug target pathways, producing rich and expressive rules.
Bean DM, 2017^16^	United Kingdom	To construct a knowledge graph containing four types of nodes: drugs, protein targets, indications, and adverse reactions	Database study	Public data on drug targets, indications and ADRs	The developed machine learning algorithm based on a simple enrichment test and first demonstrated how well this method performed at classifying known causes of adverse reactions (AUC 0.92). A cross validation scheme in which 10% of drug-adverse reaction edges were systematically deleted per fold revealed that the method correctly predicts on average 68% of the deleted edges.
Onay A, 2017^17^	Turkey	To develop computational classification methods that can distinguish between approved drugs and withdrawn ones.	support vector machines (SVMs) and ensemble methods (EMs)	6 data sets with a total of 110 approved and withdrawn drugs for all and nervous system diseases.	The descriptors number of total chemotypes and bond CN amine aliphatic generic were the most significant. On the test set for drug data set including all diseases, the developed Medium Gaussian SVM model achieved 78% prediction accuracy. For phycholeptics and psychoanaleptics drugs, the bagged tree and linear SVM models achieved 89% accuracy. In nervous system withdrawn drug (NSWD) data sets, a set of discriminative fragments was obtained.
Dandala B, 2019^18^	USA	(i) To detect mentions of medication name and attributes (dosage, frequency, route, and duration), as well as ADEs, indications, other signs or symptoms (SSLIF), and severity. (ii) To identify the characteristics of a medication, the relationships between medications and ADEs (referred to as “adverse” relations), medications and indications (referred to as “reason” relations), and the severity of an ADE, sign, or symptom. (iii) To develop an integrated system of the two tasks, in which entities recognized by the system in task 1 are used to identify relationships.	Natural language processing (NLP) techniques	1089 de-identifed clinical notes of 21 cancer patients, of which 213 were the unseen test dataset and 876 training dataset	The accuracy analysis of the three methods revealed that the joint modelling technique improved performance (F measure) by nearly 3% points (4.5% relative) over the traditional approach, and the addition of FAERS information improved system performance by another 1% point (1.4% relative)—achieving an overall F measure of 0.661.
Dey S, 2018^19^	USA	To develop machine learning models, including a deep learning framework, that can predict ADRs and identify the molecular substructures associated with those ADRs without having to define the substructures beforehand.	Database study	Shortest-path, PubChem, MACCS, CDK Standard, CDK Graph, Klekota-Roth (KR), *E*-State, CDK Hybridization, CDK Extended, and ECFP6 were the ten popular chemical fingerprints used for ADR prediction tasks.	The model's performance was compared with ten different state-of-the-art fingerprint models, the neural fingerprints from the deep learning model outperformed all other methods in predicting ADRs. Important molecular substructures were associated with specific ADRs using feature analysis on drug structures and statistically assessed their associations.
Yang X, 2019^20^	USA	To develop a machine learning-based clinical NLP system - MADEx for detecting medications, ADEs and their relations from clinical notes.	Database study	A corpus of 1089 de-identified clinical notes was used to extract clinical NER and relations.	On the validation set, the RNN-1 model outperformed the CRFs model with an F1-score of 0.8897. On the test set, RNN-2 had the highest F1-score of 0.8233, outperforming RNN-1 (0.8134) and CRFs (0.7250).
Chapman AB, 2019^21^	USA	To develop a natural language processing (NLP) system that will identify mentions of symptoms and drugs in clinical notes and label the relationship between the mentions as indications or adverse drug effects (ADEs).	Database study - Named Entity Recognition (NER)	Clinical notes from the UMASS hospital were de-identified and manually annotated into categories.	The NLP system validation was carried out against the evaluation set provided by the MADE 1.0 challenge, and the performance of our system was compared to that of other submitted systems. The micro-averaged F1 score for NER was 80.9%, RE was 88.1%, and the final system was 61.2%.
Duan L, 2012^22^	USA	To develop methods for identifying the associations that the observational medical outcomes partnership (OMOP) defined in order to simulate data from the observational simulated dataset.	Database study	The simulated dataset contains ten million people, 90 million drug exposures from 5000 different drugs, and 300 million condition occurrences from 4500 different conditions from a period of over a ten-years.	The experimental results show that the proposed pattern discovery method improves the standard baseline algorithm—chi-square—by 23.83% on the simulated OMOP dataset.
Huang LC, 2011^23^	USA	To develop a computational systems pharmacology framework consisting of statistical modelling and machine learning to predict ADR of drugs.	Database study	Clinical observation data combined with drug target data, PPI networks, and gene ontology (GO) annotations.	An in-silico model based on this framework could predict cardiotoxicity ADRs with reasonable accuracy (median AUC = 0.771, Accuracy = 0.675, Sensitivity = 0.632, and Specificity = 0.789). The findings also highlighted the importance of using prior knowledge, such as gene networks and gene annotations, to improve future ADR assessments.

Community pharmacy					
Takase T, 2022^26^	Japan	To assess the impact on medication dispensing of automated dispensing robots and collaborative work with pharmacy support staff.	Prospective study	Prescriptions filled with each dispensing device during the study periods	The total incidence of prevented dispensing errors was significantly reduced after the robotic dispensing system was introduced (0.204% [324/158,548] to 0.044% [50/114,111], *p* < 0.001). The total number of unpreventable dispensing errors was reduced significantly (0.015% [24/158,548] to 0.002% [2/114,111], p < 0.001). The number of cases of wrong strength and wrong drug, which can have serious consequences for a patient's health, had reduced to almost zero. Pharmacists' median dispensing time per prescription was significantly reduced (from 60 to 23 s, p < 0.001).

Computerized physician order entry					
Jungreithmayr V, 2021^34^	Germany	To investigate the distinct effects of a CPOE system implemented on general wards in a large tertiary care hospital on the quality of prescription documentation.	Retrospective analysis	Two groups of 160 patients' prescriptions	The overall mean prescription-Fscore increased from 57.4% ± 12.0% (*n* = 1850 prescriptions) prior to implementation to 89.8% ± 7.2% (*n* = 1592 prescriptions) after (p < 0.001). Individual criteria-Fscores improved significantly in most criteria (*n* = 14), with 6 criteria achieving a total score of 100% after CPOE implementation. While the implementation of a CPOE system generally improved the quality of prescription documentation, certain criteria were difficult to meet even with the assistance of a CPOE system.

Dose recommendation					
Blasiak A, 2022^37^	Singapore	To develop CURATE.AI, a small data, AI-derived platform that harnesses only a patient's own prospectively/longitudinally acquired data to dynamically identify their own optimal and personalized doses.	Open-label, multi-center, single-arm, prospective feasibility trial	Patients with advanced solid tumours who were treated with single-agent capecitabine, XELOX, or XELIRI (plus/minus biologics).	When compared to the projected SOC dose, the prescribed dose was reduced by 20% (13.8%) on average. The nine patients who were reported completed 3.9 cycles (2.2 cycles), with the longest participation lasting 8 cycles. CURATE. AI recommendations were considered in 27 of the 40 total dosing decisions, and 26 of those decisions were accepted for prescription.
Wang Z, 2022^39^	Singapore	To develop a machine learning algorithms to recommend vancomycin dosage in tertiary general hospital patients.	Retrospective analysis	Inpatients, who received at least one vancomycin injection during the period from January 1, 2017 to December 31, 2019, were selected.	Only a small proportion (34.1%) of current injection doses could achieve the desired vancomycin trough level (14–20 μg/ml) in the 3-year data. The machine learning models outperformed the traditional pharmacokinetic models in terms of PAR and MAE. In the test data, the model outperformed the other previously developed machine learning models.
Hu YH, 2018^40^	Taiwan	To predict the appropriateness of initial digoxin dosage using machine learning techniques.	Retrospective analysis	Patients who had been hospitalized and had their conditions treated with digoxin between 2004 and 2013	Six machine learning techniques were considered: decision tree (C4.5), kNN, classification and regression tree (CART), randomForest (RF), multilayer perceptron (MLP), and logistic regression (LGR). The area under the ROC curve (AUC) of RF (0.912) was excellent in the non-DDI group, followed by MLP (0.813), CART (0.791), and C4.5 (0.784); the remaining classifiers performed poorly. The AUC of RF (0.892) was the best for the DDI group, followed by CART (0.795), MLP (0.777), and C4.5 (0.774); the other classifiers performed poorly.
Roche-Lima A, 2020^41^	Puerto Rico	Using genetic and non-genetic clinical data, compare seven ML methods for predicting stable warfarin dosing in Caribbean Hispanic patients.	An open-label, single-center, population-based, observational, retrospective cohort study	Participants were recruited from an anticoagulation clinic affiliated with the Veteran's Affairs Caribbean Healthcare System (VACHS) in San Juan, Puerto Rico.	Random forest regression (RFR) outperformed all other methods, with a mean absolute error (MAE) of 4.73 mg/week and 80.56% of cases falling within ±20% of the actual stabilization dose. RFR performance is also superior to the rest of the models with “normal” dose requirements (MAE = 2.91 mg/week). Support vector regression (SVR) outperforms the others in the “sensitive” group, with a lower MAE of 4.79 mg/week. Finally, multivariate adaptive splines (MARS) performed best in the resistant group (MAE = 7.22 mg/week) with 66.7% of predictions within ±20%. Models generated by the RFR, MARS, and SVR algorithms predicted weekly warfarin dosing significantly better than other algorithms in the studied cohorts.

Drug-drug interactions					
Mei S, 2021^43^	China	Based on potential drug perturbations on associated genes and signaling pathways, an attempt was made to simplify computational modelling for drug-drug interaction prediction.	Database study	Only drugs that have been discovered to target at least one human gene were represented in the drug target profile.	The SP, SE, and MCC metrics on the two classes show that the proposed framework is less biased, with 0.9556 on the positive class, 0.9402 on the negative class, and 0.9007 overall MMC. These findings show that a drug target profile alone can accurately separate interacting drug pairs from non-interacting drug pairs (accuracy = 94.79%).
Van Laere S, 2022^50^	Belgium	To compare the performance of conventional statistical methods (CSM) and machine learning techniques (MLT)	Database study	Retrospective data of 512 and 102 drug-drug interactions with possible drug-induced QTc prolongation	In a hold-out dataset, random forest and Adaboost classification performed best, with an equal harmonic mean of sensitivity and specificity (HMSS) of 81.2% and an equal accuracy of 82.4%. Both sensitivity and specificity were high (respectively 75.6% and 87.7%). All CSM performed similarly, with HMSS ranging from 60.3 to 66.3%. The logistic regression overall performance was 62.0%. In terms of predicting drug-induced QTc prolongation, MLT (bagging and boosting) outperformed CSM.
Song, D, 2018^51^	China	To develop a machine learning model using support vector machines (SVMs) based on a previously reported set of similarity measures and extensive training data sets.	Database study	DrugBank provided 10,705 DDI that were associated with 1162 drugs.	The predictive performance of AUROC the 10-fold cross-validation studies was >0.97, which is significantly better than the AUROC of 0.67 of an analogously developed machine learning model. The pairwise kernel SVM model outperformed previous works in terms of accuracy, and it can be used as a pharmacovigilance tool to detect potential DDI.

Electronic Health Records					
Dalal AK, 2019^54^	USA	To describe the experience of the systems engineering (SE) and human factors (HF) core team to support individual projects during each phase of a suite of novel digital health tools integrated with the electronic health record (EHR) across the 5 phases of AHRQ's SE lifecycle: problem analysis, design, development, implementation, and evaluation	Case report	Patient Safety Learning Laboratory (PSLL) members	Of the 29 participants, 19 and 16 took part in surveys and focus groups about their perceptions of SE and HF, respectively. Over the course of the four-year project, we identified seven themes in the application of the 12 SE and HF methods. Qualitative methods (interviews, focus groups, observations, and usability testing) were the most used, typically by individual project teams, and produced the most insight. The SE and HF core teams typically used quantitative methods (failure mode and effects analysis, simulation modelling), but the results were variable.
Balestra M, 2021^56^	USA	To develop a predictive model for identifying orders that require intervention based solely on the ordering provider's interactions with the EHR.	Database study	Data from the EHR system on provider actions and pharmacy orders	In both the area under the receiver-operator (AUROC) and precision-recall (AUPR) curves, the XGBoost algorithm outperformed both logistic regressions and the random forest algorithm by a significant margin. The area under the receiver-operator characteristic curve was 0.91, and the area under the precision-recall curve was 0.44.

Potentially inappropriate medications					
Xingwei W, 2022^60^	China	To evaluate the data on potentially inappropriate prescribing (PIP), potentially inappropriate medications (PIM), and potential prescribing omissions (PPO) in elderly patients with cardiovascular disease, and to develop a prediction platform using multiple machine learning algorithms to predict the risk of PIP, PIM, and PPO in elderly patients with cardiovascular disease.	Retrospective analysis	This study included participants who were discharged from the Department of Geriatric Cardiology at Sichuan Provincial People's Hospital between January 2017 and June 2018.	The study included 404 patients in total (318 [78.7%] with PIP; 112 [27.7%] with PIM; and 273 [67.6%] with PPO). Following data sampling and feature selection, 15 datasets were obtained, and 270 risk warning models based on them were built to predict PIP, PPO, and PIM, respectively. The AUCs of the best model for PIP, PPO, and PIM were 0.8341, 0.7007, and 0.7061, respectively, according to external validation. The findings indicated that angina, the number of medications, the number of diseases, and age were the most important factors in the PIP risk warning model. The risk warning platform was developed to predict PIP, PIM, and PPO, with acceptable accuracy, prediction performance, and clinical application potential.
Tai, C.-T, 2020^61^	Taiwan	To predict the risk of high-alert medication treatment (digoxin) using machine-learning techniques	Retrospective analysis	This study included patients who had accepted digoxin therapy while hospitalized between January 2004 and December 2013.	AUC values ranged from 0.551 to 0.836. The RF classifier performed the best (0.836; excellent discrimination), followed by C4.5 (0.719) and ANN (0.688); the remaining classifiers performed poorly. This study found that machine-learning techniques can improve prediction accuracy for high-alert medication treatment, lowering the risk of ADEs and improving medication safety.
Wongyikul P, 2021^62^	Thailand	To develop a novel approach that employs machine learning models to predict the appropriateness of high alert drugs (HAD) use for a specific patient visit.	Retrospective analysis	Patient data from the Maharaj Nakorn Chiang Mai Hospital's outpatient and inpatient departments in 2018	The machine learning algorithm identified over 98% of actual HAD mismatches in the test set and 99% in the evaluation set when screening drug prescription events with a risk of HAD inappropriate use. This study demonstrates that machine learning plays an important role in screening and reducing errors in HAD prescriptions.
Patel J, 2021^63^	USA	To examine the prevalence and leading predictors of potentially inappropriate NSAIDs use among older adults with OA using real-world data from nationally representative commercial health insurance claims with the help of machine learning approaches.	Retrospective cohort study	Older adults with OA were identified using one inpatient or two outpatient claims at least 30 days apart that consisted of OA diagnosis codes (ICD-10 codes M15–M19) during the baseline year and were required that these adults be enrolled in Medicare Advantage plans with medical and pharmacy benefits during 2015 and 2016 (i.e., 24 months).	XGBoost and CVLR- both models had an AUROC value of 0.92 (95% CI: 0.91–0.93) and 0.91 (95% CI: 0.90–0.92), respectively. While both models had similar accuracy and specificity, CVLR had better precision (0.83 vs. 0.81). On the other hand, XGBoost performed better on all other metrics being compared, including recall, F1 score, and kappa statistic.

Medication adherence					
Brath H, 2013^68^	Austria	To test a remote medication adherence measurement system (mAMS) based on mobile health (mHealth) in elderly patients with high cardiovascular risk who were being treated for diabetes, high cholesterol, and hypertension.	Randomized single-blinded (doctor blinded), controlled, single centre study with crossover design	150 patients with a known risk of cardiovascular disease (Type 2 diabetes, hypertension, hypercholesterolemia)	A comparison of medication adherence in the monitoring and control phases for the four different medications revealed a significant difference in metformin intake (*P* = 0.04) favouring the MON phase. This result did not consider the two study groups separately. There was no significant difference between the other three medications.
Wiegratz I, 2015^69^	Five European countries (France, Germany, Italy, Spain, UK)	To assess the effect of an acoustic alarm function on adherence to ethinylestradiol (EE) 20 g/drospirenone 3 mg in a flexible extended regimen (EE/drospirenoneFlex) among women seeking oral contraception in five European countries (France, Germany, Italy, Spain, and the United Kingdom).	Randomized, parallel-group open-label study	Women between the ages of 18 and 35 (smokers up to the age of 30) in good general health who want contraception	Dispenser data revealed a daily delay in pill release of 88 (126) minutes in group A vs 178 (140) minutes in group B (*P* < 0.0001). The median (lower quartile, Q1; upper quartile, Q3) number of missed pills in group A was 0 (0; 1) vs 4 (1; 9) in group B (P < 0.0001). The results of the diary cards revealed similar trends; however, underreporting of missed pills was evident in both groups. During the 424 woman-years of exposure, no pregnancies were reported. The mean (SD) EE/drospirenoneFlex cycle length was 51.0 (31.8) days across the two groups, with significant regional differences, and the mean (SD) number of bleeding/spotting days was 50.4 (30.0). EE/drospirenoneFlex was well tolerated, with 80% of women satisfied with the treatment.
Wang R, 2014^70^	USA	To assess how wireless wearable devices equipped with a tri-axial accelerometer can be used to detect and classify user hand gestures during solid-phase medication administration.	Prospective observational study	Twenty-five subjects, aged 21 years and older	Using hand gesture signals, the true positive rate was 84.17% and the false alarm rate was 13.33%, demonstrating that hand gestures could be used to effectively identify pill taking activity.
Bilodeau GA, 2011^71^	Canada	To develop and test a computer vision system for monitoring medication intake in the context of home care services.	Prospective observational study	Not mentioned	Consistently low false positive and false negative values for skin detection was obtained. The algorithm struggled most with the Guillaume and PierLuc video sequences. Again, the results for face and hand tracking were generally good. TPface and TPhands had high values of 98% and 94%, respectively.
McCall C, 2010^72^	USA	To develop an economical and marketable RFID-based Medication Adherence Intelligence System (RMAIS) that will allow patients to adhere to prescribed medication schedules with minimal effort.	Prospective study	Not mentioned	By reminding a patient of the prescribed time for medication and dispensing it in a fully automatic and error-proof manner, the system is patient-centered and user-friendly. It is a novel motorized rotation platform design with the smooth integration of a scale, an RFID reader, and the rotation platform. This system also includes an Internet-based notification function that alerts the patient when it is time to take medicine and reports deviations from the prescribed schedule to primary care physicians or pharmacists.
Shtrichman R, 2018^73^	Israel	To assess ReX feasibility through human factor studies that include assessing ReX safety, acceptance, and usability, as well as ReX efficacy in providing pills according to a preprogrammed dose regimen, managing reminders and adherence data, and increasing adherence rate compared to the standard of care.	Self-controlled study	59 human subjects (29 males and 30 females) ranging in age from 18 to 92 years.	81% (48/59) of subjects rated the ReX device as simple to use. The 4-day home-use study assessed the ReX system's safety, efficacy, and usability. There was no adverse event; no pill overdose or pill malformation was reported. Overall adherence in the ReX test was 97.6% versus 76.3% in the control test (*P* < 0.001). In the event of a missed pill, real-time, personalized reminders contributed to 18.0% of doses taken during the ReX test. 87% (35/40) of subjects found the ReX system simple to use, and 90% (36/40) felt comfortable using it for medication.

Medication errors					
Segal G, 2019^79^	Israel	To assess the precision, validity, and clinical utility of medication error alerts generated by a novel system that employs outlier detection screening algorithms.	Prospective study	Patients admitted to Sheba Medical Center's single 38-bed internal medicine department	The system's alert burden was low, with alerts generated for only 0.4% of all medication orders. 60% of the alerts were raised after the medication had already been administered due to changes in the patients' status that necessitated medication changes (eg, changes in vital signs). 85% of the alerts were clinically valid, and 80 % were clinically useful. 43% of the alerts resulted in changes in subsequent medical orders.
Dos Santos HD, 2018^80^	Brazil	To develop a Density-Distance-Centrality (DDC) unsupervised method for detecting potential outlier prescriptions.	Database study	Dataset containing 21 different medications prescribed at Hospital Nossa Senhora da Conceic¸ao	When compared to other methods for detecting overdose and underdose in medical prescriptions, this approach yielded better results. Furthermore, most of the false positives detected by the algorithm were potential prescription errors.
Nagata K, 2021^82^	Japan	To detect extreme overdose and underdose prescriptions that occur very rarely in clinical practice using unsupervised machine learning algorithms.	Retrospective analysis	Retrospective analysis	The model identified 27 out of 31 clinical overdose and underdose prescriptions as abnormal (87.1%). The OCSVM models developed performed well in detecting synthetic overdose prescriptions (precision 0.986, recall 0.964, and F-measure 0.973) as well as synthetic underdose prescriptions (precision 0.980, recall 0.794, and F-measure 0.839). In a comparative analysis, OCSVM performed the best. The models correctly identified the majority of clinical overdose and underdose prescriptions and performed well in synthetic data analysis.
Yalçın N, 2023^83^	Turkey	To develop models that predict the presence of medication errors (MEs) (prescription, preparation, administration, and monitoring) using machine learning in NICU patients.	Randomized, prospective, observational cohort study	Neonates admitted to a 22-bed capacity NICU in Ankara, Turkey, between February 2020 and July 2021.	The prevalence (the ratio of drug errors) was comparable between the train and test sets (64% for the train set and 59% for the test set). The performance measures were calculated as follows: accuracy 0.919 (95% CI 0.858–0.956), sensitivity 0.918 (95% CI 0.844–0.964), specificity 0.922 (95% CI 0.829–0.973), PPV 0.944 (95% CI 0.884–0.974), NPV 0.887 (95% CI) 0.804–0.937), AUC 0.920 (95% CI 0.876–0.970), and F 1 score 0.931. A higher AUC indicated that the model correctly classified 92% of the patients as having physician- or nurse-related MEs.
Corny J, 2020^84^	France	To test the accuracy of a hybrid clinical decision support system in prioritizing prescription checks to improve patient safety and clinical outcomes by lowering the risk of prescribing errors.	Retrospective analysis	Retrospective analysis	The pharmacist analyzed 412 individual patients (3364 prescription orders) in an independent validation dataset, our digital system's areas under the receiving-operating characteristic and precision-recall curves were 0.81 and 0.75, respectively, demonstrating greater accuracy than the CDS system (0.65 and 0.56, respectively) and multicriteria query techniques (0.68 and 0.56, respectively).

Medication Therapy Management (MTM)					
Kessler, S, 2021^85^	USA	To evaluate the impact of a novel artificial intelligence (AI) platform that identifies members and provides decision support to clinicians performing telephonic interventions similar to MTM and CMM with high-risk Medicaid members on actual medical claims.	Retrospective observational study	2150 Medicaid members, primarily middle-aged (aged 40–64 years), with an average of 10 chronic condition medications among a total of 25 medications.	Receiving interventions was found to have statistically significant correlations with lower costs and utilisation. The economic study discovered a 19.3% reduction in the TCoC (*P* < 0.001), which, when applied to a preintervention monthly cost of $2872, resulted in a $554 per member per month savings (PMPM). Medication costs were reduced by 17.4% (P < 0.001), resulting in a savings of $192 PMPM when compared to the preintervention cost of $1110. The utilisation study discovered a 15.1% decrease in ED visits (*P* = 0.002), a 9.4% decrease in hospital admissions (*P* = 0.008), and a 10.2% decrease in bed days (*P* = 0.01). Based on TCoC savings and programme costs, the return on investment is 12.4:1.
Bu F, 2022^86^	China	During the COVID-19 pandemic, to establish an internet hospital pharmacy service mode based on artificial intelligence (AI) and provide new insights into pharmacy services in internet hospitals.	Prospective study	Users who benefit from Shanghai medical insurance settlement.	The AI preview qualified rate was 83.65%. Among the 16.35% of inappropriate prescriptions, 49% were accepted and modified proactively by physicians, while 51% were passed after pharmacists intervened. For collecting their medication in the internet hospital, 86% of patients preferred the “offline self-pick-up” mode, which allowed the QR code to be fully utilized. There were 426 medication consultants served, with 48.83% of them consulting outside of working hours. As a result, when pharmacists were unavailable, an AI-based medication consultation was proposed.

Telemedicine					
Schiff GD, 2019^90^	USA	To evaluate a novel interactive voice response (IVR) platform for detecting patient-reported symptoms.	Cluster randomized controlled trial	Adult primary care patients seen at Brigham and Women's Hospital and North Shore Physician's Group practices.	320 patients were transferred to the pharmacist and discussed 1021 potentially drug-related symptoms based on positive symptom responses or requests to speak with a pharmacist. Of these, 188 (18.5%) were determined to be probably related to the medication, while 479 (47.1%) were determined to be possibly related to the medication. Intervention patients were significantly more likely than control clinic patients to have adverse effects documented in the medical record by a physician (277 vs. 164 adverse effects, *p* < 0.0001, and 177 vs. 122 patients discontinued with documented adverse effects, p < 0.0001).

### 3.2 Challenges in using AI in pharmacy practice

#### 3.2.1 Data privacy and security

Concerns about data privacy and security have arisen with the widespread use of AI-based applications. Health information is sensitive and a common target for data breaches. Patient data protection is thus critical.^95^ Some patients may be concerned that their data collection will infringe on their privacy, and lawsuits have been filed in response to data-sharing between large health systems and AI developers.^96^ Patient consent is an important factor in data privacy concerns, as healthcare organizations may allow the large-scale use of patient data for AI training without obtaining sufficient individual patient consent. DeepMind Health was acquired by Google in 2018. Their application, Streams, which contains an algorithm for managing patients with acute kidney injuries, was making headlines after it was revealed that the National Health Services (NHS) had given DeepMind servers the data of 1.6 million patients in order to train its algorithm without the patient consent.^94^^,^^97^

#### 3.2.2 Bias

Biases in the data collection used to train AI models can lead to biased results.^98^ Minorities, for example, may be under-represented in datasets due to racial biases in dataset creation, resulting in lower-than-expected prediction performance. Even if AI systems are trained on accurate, representative data, problems may arise if the data reflects underlying biases and inequalities in the health-care system.^92^ For example, African-American patients receive less opioid analgesia on average than white patients; an AI system learning from health-care records may learn to recommend lower doses of opioid analgesia to African-American patients, despite the fact that this decision is based on systemic bias rather than biological reality.^99^

#### 3.2.3 Data integration

Following the acquisition of data, the next challenge is the development of AI technology. Overfitting can occur when the system learns irrelevant relationships between patient variables and outcomes. It is caused by having too many variable parameters in relation to outcomes, and as a result, the algorithm predicts using inappropriate features.^100^

Some classification and clustering algorithms may produce very good accuracy when applied to a small amount of data; however, this may not be realistic or applicable. To be used in AI techniques, the collected data must be pre-processed. Text data, on the other hand, necessitate extensive natural language processing before use. Text, numeric, image, and video data must sometimes be integrated using the same algorithm, which is one of the most difficult challenges in medical data processing.^101^, ^102^, 103. Medical data can be collected in a variety of formats and from a variety of sources, including medical images, 3D video sequences, photographs, and numeric data. In healthcare data analysis, collecting clean, robust, and efficient data is a challenge.^104^

#### 3.2.4 Patient safety

Data collected from hospitals are sometimes of poor quality or inaccurate, missing data points. This leads to data error, which is one of the most difficult challenges in medical data processing using AI.^94^ Another issue is ML algorithm decision errors, when the applied algorithm is inappropriate for the given data, or the data is not reliable enough to be used in classification algorithms such as neural networks, decision trees, and Bayesian networks.^104^

#### 3.2.5 Clinical implementation

The lack of empirical evidence proving the efficacy of AI-based interventions in prospective clinical trials is the first barrier to successful implementation. The majority of AI research in healthcare is generally retrospective, in a controlled environment. As a result, extrapolating results to real-world scenario is difficult.^105^

#### 3.2.6 Ethical concerns

The other main concern, apart from data privacy and security, is accountability. Poor decisions, particularly in healthcare, have serious consequences, and the current paradigm holds that someone must be held accountable.^106^ However, the issue of accountability becomes far more important when considering AI applications that aim to improve patient outcomes, especially when things go wrong. As a result, it is unclear who should bear responsibility if the system fails. Holding the physician accountable may appear unfair because the algorithm was not developed or controlled in any way by them, but holding the developer accountable appears too far removed from the clinical context.^94^

#### 3.2.7 Social concerns

One of the major social concerns is the AI in healthcare, will replace jobs, making healthcare workers obsolete. The threat of replacement leads to distrust and opposition to AI-based interventions in the healthcare. This belief, however, is largely based on a misunderstanding of AI in its various forms.^107^

Healthcare professionals have generally failed to keep pace with other professionals in terms of incorporating new technologies into their daily work. Previous experiences in healthcare indicate that the implementation period is an important stage in the innovation process. In practice, inventing and testing a new AI technology is not enough; other factors that can stymie its implementation in real-world healthcare, such as.^108^^,^^109^

(1) the limited data structure and quality in existing electronic health systems,
(2) the alteration of the clinician-patient relationship,
(3) the difficulties associated with clinical integration and interoperability, must also be considered.

## 4 Conclusion

By incorporating AI into clinical practice, health care professionals can augment their decision-making processes and provide patients with personalized care. AI allows for greater collaboration between different healthcare services provided to a single patient. For patients, AI may be a useful tool for providing guidance on how and when to take a medication, aiding in patient education, and promoting medication adherence and also AI may be used to know how and where to obtain the most cost-effective healthcare and how best to communicate with healthcare professionals, optimize the health monitoring using wearables devices, provide everyday lifestyle and health guidance, and integrate diet and exercise.

Clear guidelines on safe implementation and evaluation of AI technology in real world settings, as well as further research to understand the AI technology's capabilities and limitations, are required. While the optimal conditions for successful AI adoption are not yet in place, there is still room for further development of AI in healthcare. These include clinical validation of AI software and interventions through rigorous clinical trials, prospective observational studies to implement and understand the long-term impact of AI on clinical decisions, the development of ethical and privacy guidelines and frameworks by relevant bodies and organizations to protect patient data and promote transparency. AI can be used to develop more personalized treatment plans and patient engagement research, to improve both patients' experiences and empower them to actively participate in medication decisions involving AI.

We propose *"pharmacointelligence,"* i.e., the integration of AI/ ML and similar advanced technologies into pharmacy practice with the sole aim of improving patient care and safety. This being said, the concepts of AI/ ML should be incorporated into the pharmacy curriculum and stakeholders should be kept abreast of innovations in this field through continuous education. As these technologies evolve at a rapid pace, the education system for pharmacists must adapt to ensure that our profession is prepared to lead these changes in care.

## Funding

This research received no specific grant from any funding agency in the public, commercial, or not-for-profit sectors.

## Declaration of Competing Interest

The authors declare that there are no conflicts of interest.

## Data Availability

Large volumes of data, such as EHRs, pharmacy records, insurance claims records, or consumer-generated information such as fitness trackers or purchasing history are required for training the AI systems.^92^ However, the healthcare data availability a complicated issue. On an organisational level, health data is expensive,^93^ and there is an in-built aversion to data sharing between hospitals because they are considered each patient's data to be the hospital's property. Further, a single patient may visit multiple healthcare providers and change insurance companies over a period, thus leading to the splitting of the data into multiple formats. This may lead to loss of data, incomplete data, risk of errors and increased expenditure for the gathering the data.^94^
